# Should self‐administered voluntary assisted dying be supervised? A Queensland case

**DOI:** 10.5694/mja2.52634

**Published:** 2025-04-13

**Authors:** Eliana Close, Katrine Del Villar, Ben P White

**Affiliations:** ^1^ Australian Centre for Health Law Research Queensland University of Technology Brisbane QLD

**Keywords:** Suicide, assisted, Euthanasia, Euthanasia, active, Terminal care, Medicolegal

All Australian states and the Australian Capital Territory have voluntary assisted dying (VAD) laws. Medication management will be topical in these laws’ mandatory reviews following a Queensland coronial inquest into the death of a person who consumed a VAD substance prescribed for their spouse. In a decision issued on 11 September 2024, the coroner found “operational flaws” in Queensland's VAD law, declaring current self‐administration procedures “inadequate to provide for medication safety and to prevent deliberate misuse”.^1^ These findings have nationwide relevance as all Australian VAD laws permit eligible persons to self‐administer without a health practitioner present.^2^

## The coronial case

On 16 May 2023, ABC (pseudonym), an older person, died after purposely consuming a VAD substance prescribed for their terminally ill spouse.^1^ Due to the sensitive nature of the case, the coroner's report includes a ban on publishing identifying details. Accordingly, ABC, their spouse (the terminally ill patient), and adult child are referred to using neutral terms.

The circumstances leading to ABC's death are set out in Box 1.

Box 1 **Background leading to the inquest into the death of ABC (a pseudonym)**

**Table:** 

ABC (a pseudonym) was an older person who lived in regional Queensland. ABC's spouse was terminally ill and was found to be eligible for VAD. The spouse appointed ABC as their contact person. Under the *Voluntary Assisted Dying Act 2021* (Qld),^3^ a contact person is authorised to receive the VAD substance on the patient's behalf and has duties to return any unused substance after death. At the spouse's request, the Queensland Voluntary Assisted Dying Support and Pharmacy Service (QVAD‐SPS) delivered the VAD substance to ABC at their home in regional Queensland, with the usual instruction booklet for how to administer it. The terminally ill spouse planned to self‐administer the VAD substance at home, but the couple both contracted COVID‐19 and were hospitalised. ABC recovered and was discharged, but the spouse remained in hospital in very poor health. The spouse lost the ability to swallow and revoked their self‐administration decision, electing instead for practitioner‐administered VAD in hospital. After the spouse died, ABC was overcome with grief and “quite unable to function”.^1^ ABC's adult child made an urgent appointment with ABC's general practitioner to discuss ABC's mental health. ABC did not return the VAD substance within the legally required 14 days after the spouse chose practitioner administration. Two days after this 14‐day period expired, QVAD‐SPS contacted ABC and ABC's adult child about return of the unused VAD substance. Four days after that, ABC's child returned home from the general practitioner to find ABC dead in a chair, after ABC had consumed the VAD substance.

ABC = pseudonym for the spouse of the terminally ill patient; COVID‐19 = coronavirus disease 2019; VAD = voluntary assisted dying.

The coroner's inquest investigated whether:

- the Queensland Statewide Support and Pharmacy Service (QVAD‐SPS) personnel had appropriately sought return of the unused VAD substance; and
- the process for self‐administration of VAD could be made safer.

The coroner heavily criticised aspects of the self‐administration framework. In particular, he was concerned that:

- the law permitted two VAD substances to be issued for one person (ie, the oral self‐administration VAD substance did not need to be returned before a second VAD substance was used in intravenous practitioner administration);
- QVAD‐SPS could not compel return of the VAD substance; and
- the self‐administration VAD substance was not in the possession of a health practitioner.

The coroner recommended that self‐administration should be supervised by a health practitioner, an option that was considered by the Queensland Law Reform Commission (prompted by a proposed VAD bill)^4^ but was not adopted.^5^

The coroner warned of “[f]urther calamity and heartbreak” for patients and families without system reform.^1^ He specifically confined his critical remarks to the system rather than the individuals working within it, noting that QVAD‐SPS personnel had not breached the law or any protocol.

## Self‐administration under Australian VAD laws

The Australian model of VAD is characterised by narrow eligibility criteria and numerous safeguards.^2^ One of these safeguards is that only medical practitioners (and in some states, nurses or nurse practitioners) who complete mandatory training and meet additional experience and expertise requirements can participate in key aspects of VAD (“VAD practitioners”). Two independent VAD practitioners assess whether a person is eligible for VAD (in the states, only medical practitioners can do VAD assessments; but in the ACT, one practitioner can be a nurse practitioner). If the person is eligible, the lead VAD practitioner (“coordinating practitioner”) writes the prescription for the VAD substance.

Australian VAD medication protocols are not publicly available but, as in other countries, the VAD substance is a combination of medications used in health care settings (including a Schedule 8 [S8] medicine).^3^, ^6^ The medication protocol differs depending on the method of administration. Self‐administration involves mixing a liquid that a person drinks (or ingests via nasogastric tube), while practitioner administration typically involves intravenous injection.^6^

Victoria and South Australia require self‐administration unless the person cannot swallow or digest the substance.^2^ Other jurisdictions allow more choice and have much higher rates of practitioner administration (Box 2).^2^, ^7^ No Australian VAD laws require supervised self‐administration, although Tasmania's law has this as an option.^2^

Box 2 **Legal framework and rates of voluntary assisted dying (VAD) method of administration by jurisdiction**

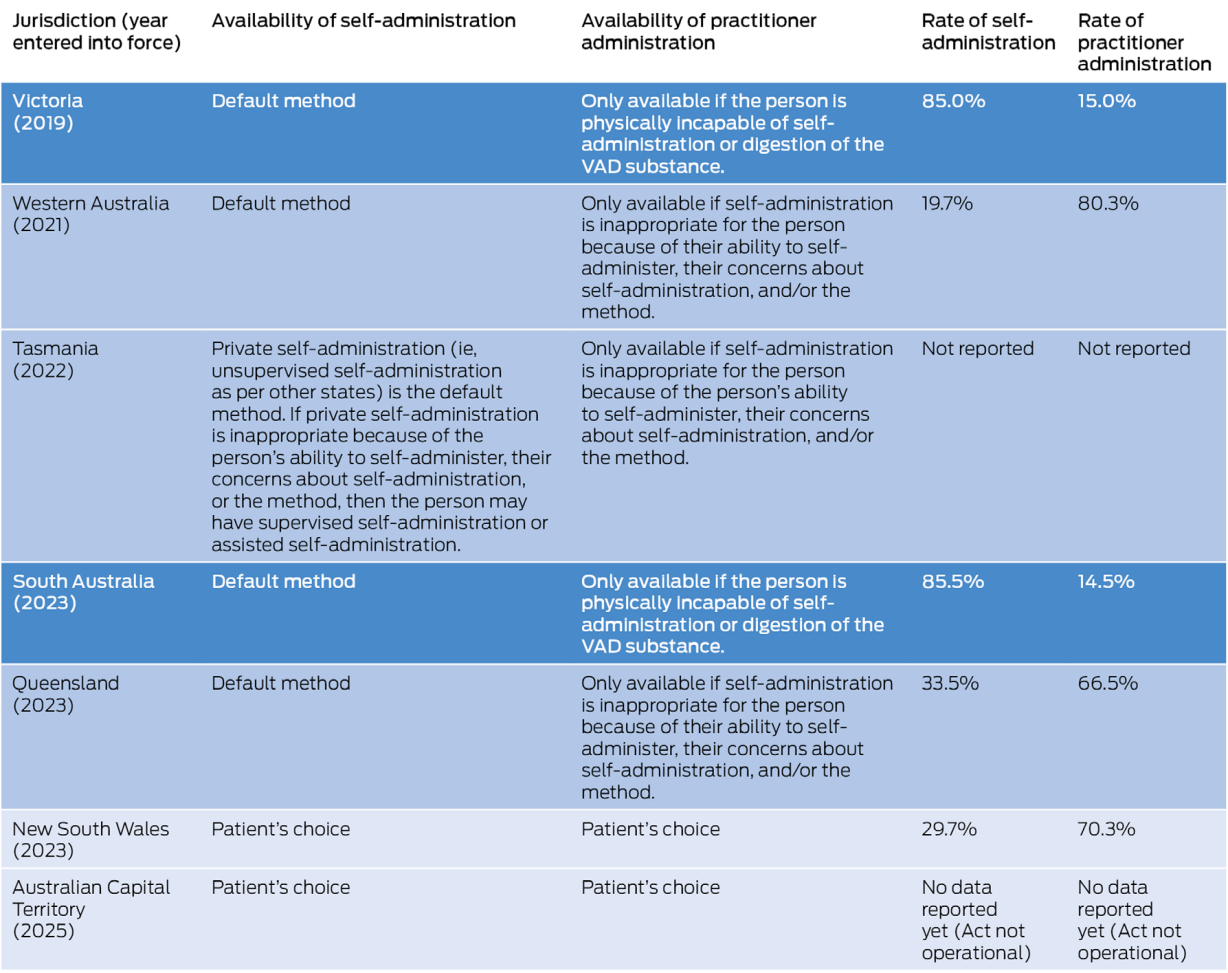

Dark blue denotes jurisdictions that permit the least choice regarding method of administration; medium blue indicates jurisdictions that provide some choice; and light blue indicates jurisdictions with free choice. Data on rates of administration are aggregated from state Voluntary Assisted Dying Review Board reports.^7^

## How is self‐administration operating in practice?

Nationally, there were 2467 VAD deaths since 2019; 1258 (51%) involved self‐administration.^7^ More eligible people were issued the VAD substance for self‐administration but chose not to take it.^7^ The ABC case is the sole instance of a person taking a VAD substance not prescribed for them.

Compliance with legal requirements to return the VAD substance has been high. To date, 17 cases involved a delay in returning the VAD substance: 12 from Victoria (between 2019 and 2024);^7^, ^8^ four from Western Australia (between 2021 and 2023);^7^ and one from Queensland (the ABC case).^1^

In the ABC case, the coroner heard evidence of reports of complications after self‐administration, including vomiting and an extended period to death.^1^ Data on VAD complication rates is limited.^7^ In its 2022–2023 annual report, Victoria's VAD Review Board stated that it had reviewed self‐administration cases with a prolonged time to death, primarily involving persons with neurodegenerative diseases causing autonomic system failure.^9^ There are no reports of the VAD substance not causing death.

## Should VAD laws require self‐administration to be supervised by a health practitioner?

### Reasons to change the current system: community safety is paramount

**Minimise community risk**. Self‐administration supervised by a VAD practitioner or other health practitioner would enhance community safety because the VAD substance would remain in a health practitioner's custody, considerably minimising the risk of diversion. As the coroner noted, the VAD substances are prescribed in large quantities to end life and are accompanied by explicit instructions on taking them, virtually guaranteeing death.

**Ensure patient has capacity**. Supervision would enable a VAD practitioner to ensure the patient has capacity at the time of self‐administration, as is required for practitioner administration.^4^ This would uphold the legislation's goals and make the framework for both methods of administration consistent.

**Provide patient with assistance if required**. Supervision would enable prompt medical assistance in the rare instance that complications occur, and may also provide additional comfort for the patient and family. ABC had expressed anxiety about mixing the VAD substance and wanted a health practitioner to be present. Currently, health practitioners sometimes attend self‐administration at the patient's request.^10^

### Reasons to retain the current system: balancing safety with broader considerations

**Impact on patient access to VAD**. Requiring self‐administration to be supervised is likely to impact some patients’ access. Currently, the person can possess the VAD substance at home to take when they choose. This can be empowering and alleviate suffering, even if a person does not ultimately proceed with VAD.^7^ Requiring VAD to be supervised makes administration dependent on health practitioner availability, which may cause delays for all patients, but particularly those in remote and rural areas. In some cases, delays may mean a person loses their opportunity for VAD because they lose capacity or die before a health practitioner is available. Requiring supervision will also exacerbate the existing shortage of willing VAD practitioners and associated delays.^7^, ^11^

**Burdens on clinicians**. Supervised self‐administration will ask more of VAD practitioners, who already have heavy workloads and lack adequate remuneration, potentially creating further system sustainability issues.^7^, ^12^ Supervised self‐administration may result in more people choosing practitioner administration, given the primary benefit to the patient of self‐administration (to choose precise timing) is diminished. As the coroner noted, VAD practitioners may be asked to supervise patients outside of typical hours (eg, at sunset or on the weekend),^1^ which would further stretch an already busy workforce. Burdens on clinicians would be intensified in jurisdictions with a small VAD workforce.

**VAD substances and other dangerous medications in the community should be managed consistently**. The coroner noted that dangerous S8 medications are subject to strict control in hospitals and health care institutions. However, they also exist unsupervised in the community in the homes of terminally ill persons receiving community palliative care. Although S8 medications can cause death,^13^ ongoing community access is permitted. This represents a policy decision based on quality of care and timely access to medication. When the risk of diversion is considered high, additional safety measures, including a locked box and limited supply, are implemented. Additionally, there may be other substances and poisons kept at home (eg, solvents), which, if consumed in sufficient quantities, would lead to death.

**A single case does not warrant significant system reform**. While the ABC case is undoubtedly tragic, this is an isolated incident of unlawfully taking a VAD substance without authorisation. It is the only recorded case of taking another's VAD dose in 1258 self‐administration cases nationally since 2019.^7^ Further, the case for reforming regulation must justify the additional financial and human resourcing implications of this change.

## Weighing evidence and competing values

Whether to require self‐administration of VAD to be supervised is a policy choice informed by weighing evidence and competing values.^14^ A policy position that prioritises community safety and emphasises protecting human life would support the coroner's view that the system's goal must be “precisely 100% compliance and that no innocent, nor unintended, person is in any way harmed”.^1^ With this view, constraints on patient autonomy to choose the timing of one's death, delays, the likelihood that some patients will be unable to access VAD, and increased burdens on practitioners, are necessary corollaries of prioritising patient and community safety and avoiding the risk of death by unlawful use of a VAD substance.

Alternatively, a policy position that takes a more pragmatic lens aims to balance safety with the burdens of supervised self‐administration and accepts that adverse outcomes sometimes occur in health care. This position accepts the relatively remote risks of permitting private self‐administration. As the Australian Medical Association Queensland stated, “a one‐off event or death did not necessarily mean the laws were terrible or needed fundamental reform”.^15^ This position is also informed by impacts on the system and individual practitioners. Occasional adverse outcomes are an unavoidable consequence of having a VAD system that prioritises patient choice and access and considers the broader impacts of supervised self‐administration on the VAD system and workforce. This policy position focuses on measures to mitigate risks, rather than eliminating them completely.

Whatever policy option is chosen, the government must ensure that it is adequately resourced. Experience with health care systems generally has shown that under‐resourced systems with a poorly supported workforce can fail to deliver safe and high quality care. Hence, if supervised self‐administration is preferred on the grounds of patient and community safety, there is a duty to provide any additional resourcing required. We urge governments to closely consider the ABC case in their VAD reviews and offer several recommendations for consideration (Box 3).

Box 3 **Recommendations when reviewing voluntary assisted dying (VAD) self‐administration laws**

**Table:** 

Reviews should consider available evidence about safety and autonomy as well as impacts on patients, families, practitioners and the system. Reviews should engage carefully and transparently with the normative questions of how to strike the balance between safety, autonomy and other considerations. If current self‐administration laws are retained: ‣ Governments should consider measures that reduce harms associated with private self‐administration, including the coroner's recommendation that the self‐administration kit must be returned before practitioner administration. Immediately after ABC's death, this practice was implemented as QVAD‐SPS protocol in Queensland (personal communication, Professor Liz Reymond, Director, QVAD‐SPS). It was subsequently incorporated into the law on 1 July 2024, through an amendment to the Voluntary Assisted Dying Act Regulation 2022 (Qld). It is also required by VAD legislation in some other states (eg, Victoria, South Australia). ‣ Governments should consider introducing policy or guidelines that, where feasible, encourage health practitioners to offer to be present for self‐administration if the patient desires. QVAD‐SPS changed its practice to always offer this shortly after the *Voluntary Assisted Dying Act 2021* (Qld) took effect based on some patients’ preferences (personal communication, Professor Liz Reymond, Director, QVAD‐SPS). Offering this is also the current practice of some health practitioners. ‣ Consideration should be given to issues raised by the coroner regarding the appointment of contact persons. One option is for governments to consider introducing or amending policy or guidelines to mitigate potential risks regarding contact persons. These may include instructions to encourage the patient to carefully choose a contact person, and procedures for care navigators or practitioners to gauge possible vulnerability or risks of diversion. If supervised self‐administration is made law, governments should provide sufficient resources to address access issues for patients (including rural and remote patients) and burdens on individual health practitioners and the system. VAD practitioners should be appropriately compensated for any after‐hours work.

ABC = pseudonym for the spouse of the terminally ill patient; QVAD‐SPS = Queensland Voluntary Assisted Dying Support and Pharmacy Service; VAD = voluntary assisted dying.

## Open access

Open access publishing facilitated by Queensland University of Technology, as part of the Wiley – Queensland University of Technology agreement via the Council of Australian University Librarians.

## Competing interests

Ben White has been engaged (with colleagues) by the Victorian, Western Australian and Queensland governments to design and provide the legislatively mandated training for health practitioners involved in voluntary assisted dying in those states. Eliana Close and Katrine Del Villar were employed on these projects. Ben White has also received funding from state governments for voluntary assisted dying research. In addition, he (with a colleague) developed a model bill for voluntary assisted dying for parliaments to consider, which was discussed in the coronial case on ABC that this article examines.

## Provenance

Not commissioned; externally peer reviewed.
